# Dementia diagnosis inequality between high‐income countries and Brazil: how challenging the diagnosis can be

**DOI:** 10.1002/alz70860_105881

**Published:** 2025-12-23

**Authors:** Isabela Resende Silva, Pedro Henrique Dutra Corrêa, Vinicius ribeiro Jeunon, Breno José Alencar Pires Barbosa, Jose Wagner Leonel Tavares‐Júnior, Bruno Iepsen, Mari Nilva Maia da Silva, Raphael Machado Castilhos, Sonia Maria Dozzi Brucki, Wyllians Vendramini Borelli, Leonardo Cruz de Souza, Elisa de Paula, França Resende, Adalberto Studart Neto, Paulo Caramelli

**Affiliations:** ^1^ Universidade Federal de Minas Gerais, Belo Horizonte, Minas Gerais, Brazil; ^2^ Universidade Federal de Minas Gerais, Belo Horizonte, Minas gerais, Brazil; ^3^ Cognitive and Behavioral Neurology Unit ‐ University of São Paulo, São Paulo, São Paulo, Brazil; ^4^ UNIVERSIDADE FEDERAL DO CEARÁ, FORTALEZA, Ceará, Brazil; ^5^ Universidade de Fortaleza, Fortaleza, Ceará, Brazil; ^6^ erviço de Neuropsiquiatria, Hospital Nina Rodrigues, São Luís, Maranão, Brazil; ^7^ Hospital de Clínicas de Porto Alegre, Porto Alegre, Rio Grande do Sul, Brazil; ^8^ University of São Paulo School of Medicine, São Paulo, São Paulo, Brazil; ^9^ Universidade Federal do Rio Grande do Sul, Porto Alegre, Rio Grande do Sul, Brazil; ^10^ Federal University of Minas Gerais, Belo Horizonte, Minas Gerais, Brazil; ^11^ University of São Paulo Medical School, São Paulo, Brazil; ^12^ Faculty of Medicine ‐ Federal University of Minas Gerais, Belo Horizonte, Minas Gerais, Brazil

## Abstract

**Background:**

Early diagnosis is essential for effective dementia management, but LMICs, including Brazil, face a significant disparity in terms of resources for diagnosis compared to high‐income countries. Therefore, we aim to assess diagnostic methods and clinical practice tools available in specialized dementia care centers across Brazil.

**Methods:**

We developed an online survey to get information about the current infrastructure, cognitive tests utilized, the availability of cerebrospinal fluid (CSF) and imaging biomarkers, and patients’ sociodemographic data. This survey gathered responses in July, 2024 from neurologists affiliated with the Brazilian Academy of Neurology who work in centers specialized in dementia management.

**Results:**

Neurologists from 24 outpatient clinics across 10 Brazilian states participated. Most clinics (87%) are associated with the Public Health System. Regarding research infrastructure, 39.1% lack a research database, and clinical data remain in medical records. All settings record age, sex, and education, but the settings often omit ethnicity (67.0%) and socioeconomic status (27.0%). Clinical dementia diagnoses are registered electronically in 78.0% of centers. Cognitive assessment relies on the Mini‐Mental State Examination (100%), the Montreal Cognitive Assessment (66.7%), and the Brief Cognitive Screening Battery (54.2%). Functional assessment relies on the Pfeffer Questionnaire in 33.3% and on the KATZ Scale in 20.8% of settings. Neuropsychiatric evaluations occur in 58.3%, especially based on the Geriatric Depression Scale. Only 29.2% of centers assess dementia staging, and Clinical Dementia Rating is the primary tool. Neuropsychological and functional evaluations are unavailable in 33.3% and 66.7% of settings, respectively. Only 29.2% assess Alzheimer's disease CSF biomarkers, differing from the routine laboratory tests (100%) and routine CSF analysis (79.2%). Brain MRI is universally available, but PET‐FDG (25%) and PET‐amyloid (12.5%) are scarce. Genetic testing is unavailable in 70.8% of clinics.

**Conclusion:**

The survey highlights significant heterogeneity and gaps in dementia diagnostic process in Brazil. Clinical and cognitive assessment dominate the diagnostic practice, contrasting with the latest criteria emphasizing biomarkers. Efforts to harmonize dementia diagnostic practices, standardize data collection and expand access to advanced diagnostic tools will enhance the dementia care landscape.

